# A graphene-on-silicon photodetector for low penetrating radiation

**DOI:** 10.1038/s41598-025-33880-0

**Published:** 2026-01-11

**Authors:** Neil Moffat, Jose Alfonso Soto Oton, Gemma Rius, Enric Cabruja, Giulio Pellegrini

**Affiliations:** 1 Instituto de Microelectrónica de Barcelona (IMB-CNM-CSIC), Cerdanyola del Valles, 08193 Barcelona, Spain; 2 Instituto de Física Corpuscular (IFIC), Parc Científic de la Universitat de València, Valencia, Spain

**Keywords:** Materials science, Nanoscience and technology, Optics and photonics, Physics

## Abstract

We introduce an innovative graphene-on-silicon photodiode designed for low penetrating radiation. Its standout feature lies in its remarkably-thin dead layer in the entrance window, setting it apart from existing photodetectors. Conventional photodetectors suffer from sensitivity limitations in the low wavelength or energy, respectively, for light or particles, due to their shallow penetration depth. Most conventional photodiodes employ a junction implant which suffers from recombination of low-penetrating photons/particles within the dead layer. Instead, we utilise the nearly transparent properties of single-layer graphene to create a depletion layer that minimises the dead layer. We combine a single junction ring (highly doped $n^{++}$ bias ring) with single-layer graphene. The graphene acts as a field plate, extended over the junction ring and covering the entire entrance window (5$\times$5 $mm^2$ active area), while being electrically isolated by an ultrathin, high K dielectric layer. In operation, the photodiode undergoes depletion upon applying a reverse bias as expected, which primarily occurs within the region beneath the field plate. We conducted Transient Current Technique measurements as the best method to assess the charge collection uniformity of the device. Remarkably, the results reveal a consistent total **100% uniformity** across the entire detector area. Nevertheless, while the collection time is position-dependent, increasing as the laser incidence point moves farther away from the bias ring, responsivity measurements show excellent response in both the deep ultra violet and vacuum ultra violet regions with $\ge$ 100% external quantum efficiency at wavelengths below 150 nm.

## Introduction

The significance of detecting low penetrating radiation can be found across a range of disciplines involving low penetrating particles, low energy X-rays (often called soft X-rays) and ultraviolet (UV) light. UV radiation, characterized by wavelengths spanning 100-400 nm and energy distribution ranging from 3.1–12.4 eV, falls under the generic term UV^1–5^. The interest in UV detectors continues to grow considerably in relation to applications in both military and civilian domains. They play pivotal roles in UV communication, industrial processes, space exploration, defense warning systems, environmental monitoring, and healthcare. Sensors sensitive in the vacuum ultraviolet (VUV) range play a crucial role in experiments detecting the scintillation light emitted by noble gases, such as liquid argon or xenon, which are widely used in large-scale detectors including DUNE^6^ and similar experiments.

Silicon has emerged as a predominant semiconductor material for UV radiation detectors, primarily in view of its favorable bandgap, minimal surface state density, established manufacturing processes, and rapid detection capabilities. Nevertheless, silicon photodetectors still grapple with a significant hurdle in the UV range: low photo-responsivity, typically registering below 0.2 A/W for $\lambda$ < 400 nm^7^. This limitation arises from the high reflectivity and shallow penetration depth of UV light within silicon. For instance, a standard silicon PN junction is typically 200 nm in depth. However, UV light penetration in silicon is limited to approximately 20 nm for wavelengths below 370 nm, resulting in photo-induced charge carriers being predominantly generated near the silicon surface. These carriers must subsequently diffuse over distances of roughly 100 nm to reach the junction region, a process that induces substantial carrier recombination and consequently constrains overall device performance. To mitigate these limitations, recent advances such as delta doping have been proposed to enable the formation of shallow junctions in conventional PIN diodes^8^. This technique offers advantages including rapid response times on the order of tens of nanoseconds and enhanced signal-to-noise ratios (SNR); however, challenges remain in addressing the effects of the dead layer associated with mid-UV and far-UV absorption.

Yet, technological strides have been made recently in silicon-based UV detection. For example, the photoconductive UV detector^9^ offers simplicity in fabrication and exhibits high gain, although it contends with notable drawbacks such as elevated dark current and sluggish response times. Schottky diode UV detectors^10^ represent an advantageous alternative with their superior responsivity, high quantum efficiency, rapid response, and operation without bias. Nevertheless, their photoactive region is confined by the substantial absorption coefficient of the metal electrode.

To achieve a high-performance silicon detector for low penetrating radiation, there is a pressing need for an ultra-shallow junction that combines efficient charge collection with swift operation. Semi-transparent metal/Si Schottky structures offer a partial solution, but they fall short in fully meeting the requirements. This is because a significant portion of the incident radiation is either reflected or absorbed by the metal layer, failing to contribute to the photocurrent and resulting in reduced photo-responsivity o detectable signal.

One promising route to overcome low responsivity in the UV is to combine nanostructured surfaces with optimized junction engineering. For example,^11^ demonstrated a boron-implanted black silicon photodiode that attains near-ideal responsivity from 200 nm up to 1000 nm, while keeping dark current, rise time, and sensitivity on par with commercial devices. Their strategy mitigates reflectance losses and surface recombination by using a textured (black Si) surface plus a carefully engineered implanted junction, effectively extending the device’s usable spectral range with minimal tradeoffs.

Moreover, the concept of using induced (or field-effect) junctions rather than traditional p–n doping can further push performance. In^12^ the authors realized a black silicon photodiode in which a negative charge in an alumina layer forms an inversion layer in n-type Si, acting as a collecting junction. Because the inversion layer is induced rather than physically diffused, recombination near the surface is suppressed, and they report an external quantum efficiency (EQE) exceeding 96% over the broad band 250–950 nm. Their device also retains good angular acceptance (up to $70^\circ$) and low reflectance via the black Si texture.

An optimal detector must exhibit not only high sensitivity, stability, and operational speed but also maintain a favourable signal-to-noise ratio.

## Materials and methods

### Detector concept

Graphene on Silicon (GoS) photodetectors have been demonstrated in prior studies, wherein the graphene is integrated onto the active silicon bulk. This configuration utilizes a Schottky contact, thus yielding Graphene/Si (G/Si) Schottky junctions, as shown in the literature, such as the pioneering research by Riazimehr^13^. Recent investigations have shown a difference in response in terms of the photocurrent between graphene-on-insulator^14^ (GIS) substrates and G/Si substrates, actually with profound implications arising from the combination of both G/Si and GIS regions within a single device.

One important point is that while reverse biased, the silicon bulk region beneath the graphene layer fails to fully deplete due to the emergence of an inversion layer, thereby emulating the response of a conventional metal-oxide-semiconductor (MOS) structure. However, under the influence of incident light of shorter wavelengths (< 600 nanometers), it is found that the photocurrent generated beneath the GIS region surpasses that obtained from the G/Si region. This phenomenon, while holding promise for deep ultraviolet (DUV) detection, introduces constraints when employed as an imaging detector, as a result of the incomplete depletion of the bulk.

We present, first, the conceptualization of a photodetector for optimal sensitivity to low penetrating radiation, $\ll$ 1 $\mu$m. The device also features a rapid photocurrent response characterized by a rise time in the range of 1-10 nanoseconds.

**Fig. 1:**
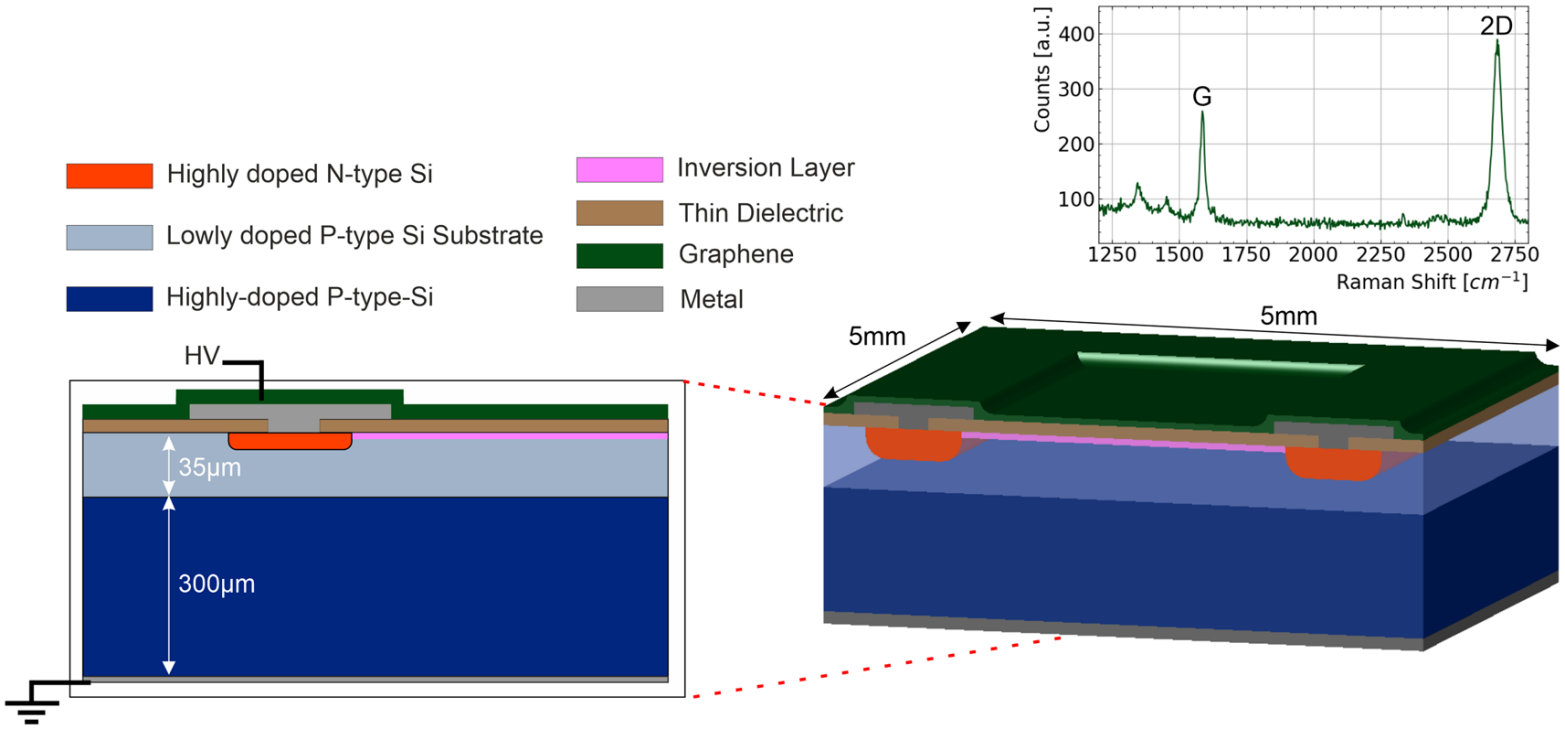
Basic schematic of the GoS device, 3D view, bias ring (orange), metal contact (grey) and graphene (green) and insert shows the cross section, with an $n^{++}$ bias ring and graphene contact on top of a thin dielectric layer (faded brown). The subsequent inversion layer is shown in pink. The top right image shows the raman spectroscopy, showing clearly the existence graphene. (Not to Scale).

Illustrated in Fig. 1, the photodetector boasts an ultra-thin entrance window comprising single-to-few graphene layers (green colour), below 1 nm in thickness. The graphene film lies on top of a high-$\kappa$ dielectric layer with a nominal thickness of 3 nm. The dielectric (faded brown colour) serves as a critical spacer component, establishing an electrically-isolated region that physically separates the contact material (graphene) and the active silicon bulk. Notably, the use of a high-$\kappa$ dielectric material is key for enabling the ultra-thin layer while upholding exceptional electrical properties, as shown in^15^.

To overcome these limitations, we have devised an improved variation of this hybrid structure, which specifically produces a detector which can be operated while fully depleted. Silicon detectors were fabricated at wafer scale as follows. A 4-inch p-type silicon wafer comprising a 35 $\mu$m high-resistivity (>5 k$\Omega$ $\cdot$cm) device layer bonded to a 300 $\mu$m low-resistivity handle wafer was used as substrate. A 4000 Å thick thermal silicon dioxide was first grown, after which photolithography was performed to define the P-stop regions for a low-dose boron implantation (Dose of $7.5 \times 10^{12} at/cm^{2}$). A second photolithography step defined the bias-ring layout, followed by a high-dose phosphorus implant (Dose of $1 \times 10^{15} at/cm^{2}$). Dopant activation and defect recovery were achieved through a high-temperature anneal at 1100 $^\circ$C, yielding a 700 Å thick additional oxide layer on the top surface. The whole wafer was then passivated by depositing a 1175 Å silicon nitride layer by LPCVD , and the oxide–nitride stack was selectively etched to open a 5$\times$5 mm$^{2}$ which defines the detector active region. A nominally 3 nm thick $HfO_2$ film was deposited by atomic layer deposition to form the high-$\kappa$ dielectric, after which a Ti/Pt metal stack (20 nm/100 nm) was patterned by evaporation and resist lift-off. The wafer was saw diced and cleaned with acetone, isopropanol and blown dry in N2. CVD graphene grown on Cu by Graphenea (San Sebastiáxn, Spain) was transferred by PMMA-assisted wet method^16^ onto the single-diode active region to complete the GIS device structure. A cut large enough to cover around 80% of the active area including electrical contact with external metal ring was used (about 6$\times$5 mm$^{2}$). Because of diode passivation and topography not all the active area coverage with transferred graphene was attempted, to minimise or prevent detachment and tearing during wet processing and ease conformality, reduce wrinkles and mechanical contact of graphene at the active region as well.

Our alternative detector concept underpins our innovation, whereby we establish electrical connectivity between the graphene conducting layer and a biasing ring encircling the outer periphery of the diode, as depicted in Fig. 1. The fabrication of the bias ring involves a process involving implantation followed by a high-temperature annealing step, conforming to standard practices observed in numerous radiation detectors.

While the schematic in Fig. 1 illustrates an $n^{++}$ bias ring, it is important to emphasize that this new conceptual design is adaptable for use with a $p^{++}$ bias ring. Importantly, our device configuration overcomes the need of realizing an ideal G/Si Schottky junction.

Under reverse bias conditions, as conventionally used in operation, the detector depletes under both the graphene layer and the bias ring, with the region beneath the graphene layer effectively functioning as a field plate, as shown in reference^17^. This configuration is key to promote the efficient collection of charges at the $n^{++}$ contacts.

**Fig. 2:**
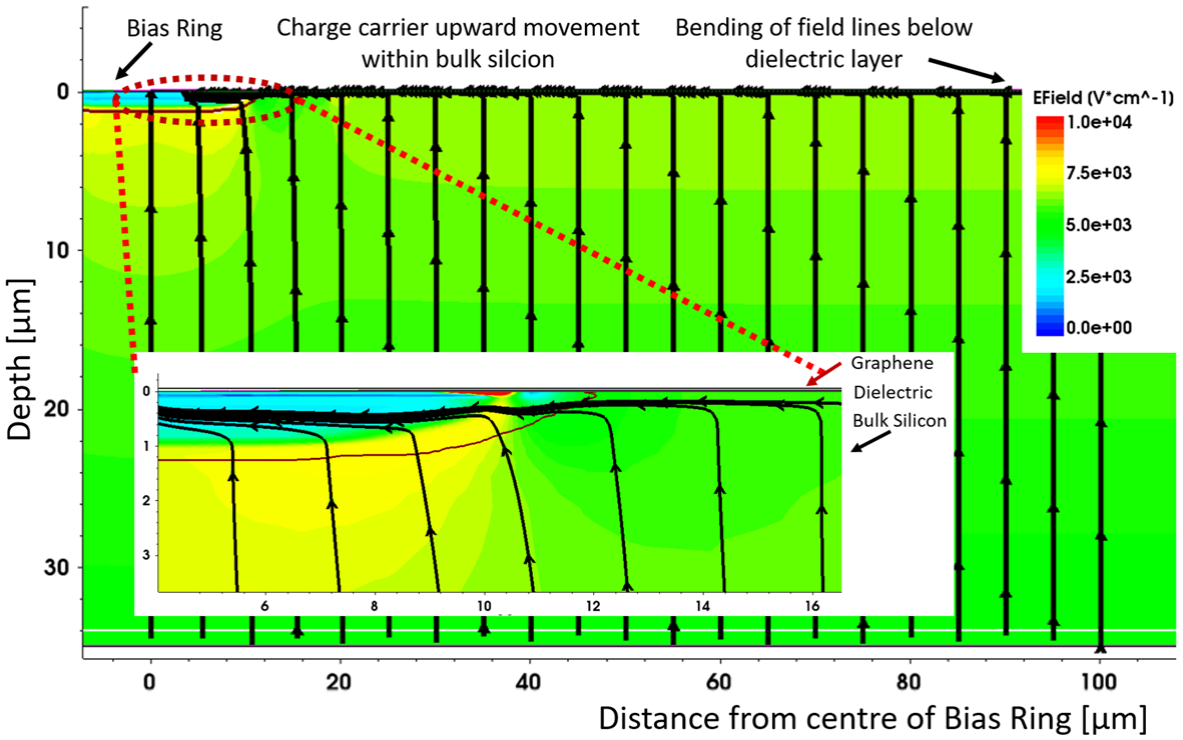
Electric field lines at the surface of the device. The field lines bend as they reach the surface towards the bias ring. The dark red line below the bias ring indicates the area around the junction implant. The inset shows the region close to the junction implant. The field lines bend towards the junction before the surface up to the edge of the implant (10 $\mu$m). Beyond this, the field lines bend at the surface.

Additionally, the graphene layer, serving as the conducting plate, shares the bias potential via both mechanical and electrical contact with the metallic ring. Advanced simulation models show the swift collection of electrons generated following interactions with particles or photons within the bulk, described in simulation section of this article, shown in Fig. 2. These electrons drift perpendicularly towards the surface due to a parallel plate electric field originating at the site of incidence. As these electrons reach the silicon-oxide interface, they cannot tunnel through the high-quality insulating layer. As they reach the interface they are deviated by the effect of a lateral electric field, resulting in a sudden bending of their trajectory toward the $n^{++}$ ring where they can be collected.

In other words, generated electrons travel without recombination across the active area, positioned immediately beneath the dielectric layer, until they are collected at the bias ring. The collection time for these generated charges remains within the narrow temporal window of a few nanoseconds. The temporal variation is contingent upon factors such as the initial pair generation position, including the laser incidence point as it will be shown below with our experimental results and in^18^.

An advantage inherent to this geometric configuration is an intrinsically low reverse current, measured within the nanoampere range. Reducing the noise of the collected signals, represents a substantial advancement compared to alternative Schottky junction devices predicated on G/Si, as substantiated in^13,19–21^. It should be noted that graphene could be substituted with any thin conductive layer, provided that, as in this case, it’s near-transparent nature is maintained.

## Methods

### Device simulation

Our simulations are designed to provide an precise representation of the actual photodetector. Sentaurus TCAD^22^ served as our primary simulation tool, which complemented by PSPICE^23^ models allowed us to construct a comprehensive model that precisely describes the operational behaviour of the device. Graphene is not an available material in the TCAD toolkit, therefore we opted for a solution which uses doped polysilicon as a surrogate to represent the electrical characteristics of graphene. To ensure a more precise quantitative representation of graphene’s distinctive physical properties, we fine-tuned the model parameters for the polysilicon material, aligning them with the fundamental characteristics of an ideal graphene layer, as specified in Table 1^24^.

**Table 1:** Material parameters of Graphene used for model^24^.

Parameters	Units	Description	Value
$E_G$	eV	Bandgap	0
$\chi$		Permittivity	25
$\mu _n$	$cm^{2}/Vs$	Electron mobility	10,000
$\mu _p$	$cm^{2}/Vs$	Hole mobility	10,000
X	kg/mole	Affinity	4.248
$v_{sat}$	*cm*/*s*	Electron saturation velocity	$4\times 10^7$

In line with the configuration and operational principles of our device, we developed a cross-sectional 2D model to precisely capture the physical characteristics and behaviour of the photodetector, as shown in Fig. 2. This model featured a p-type substrate with dimensions of 35 $\mu$m in thickness and 2500 $\mu$m in width, characterized by a doping concentration of $1 \times 10^{12}~cm^{-3}$. The rear aspect of the device was uniformly doped with $p^{++}$, while on the front side, an isolated $n^{++}$ bias ring was positioned. The $n^{++}$ bias ring was electrically connected to the high-voltage (HV) contact, overlapping the ring structure. A thin dielectric layer, 3 nm thick ($HfO_{2}$), was deposited on the surface of the device, while the $p^{++}$ region is grounded. Subsequently, a large-area graphene film was conformally deposited on top of the contact and oxide layer, spanning the entire surface area of the device, equivalent to 2500 $\mu$m. Notably, the simulation domain represents precisely half of the actual device, with symmetry assumptions facilitating the representation of electrical behaviour.

Our simulation process applies the quasi-stationary mode, a well-established technique for assessing the current-voltage characteristics of semiconductor devices. This approach involves solving the simulation of the device operation under steady-state conditions. Subsequently, incremental adjustments to the electrode voltages are introduced, each by a small increment, and the device is then resolved in this new steady state. This iterative cycle continues until a predetermined set-point voltage, mirroring the experimental applied bias.

The simulation was set to a apply a reverse bias to the bias ring while holding the $p^{++}$ contact to ground. As the applied voltage is progressively increased, the depletion region within the bulk of the photodetector also extends until reaching full depletion, at approx 2 V. This distinct behaviour represents a departure from the response typically observed in graphene-on-insulating substrate (GoS) structures reported in prior research by Riazimehr^13^ and Hafsi^25^. Importantly, this plausible deviation is understood and implies that the entire depth of the detector’s bulk remains active, allowing the charge generated throughout the bulk’s entire thickness to be collected. Consequently, thick detectors can be produced with the GoS structure while featuring lower capacitance or lower noise on one side of the detector and a pixellated structure on the other side, which makes them ideal for imaging.

The most intriguing observation from our simulation is the orientation of electric field lines. Initially perpendicular to the surface of the detector, these field lines exhibit a abrupt deflection within a very thin region underneath the surface, assuming a perpendicular orientation to the surface and directing towards the $n^{++}$ bias ring, as visually depicted in Fig. 2. This phenomenon implies that any charge generated within the detector must first traverse a vertical trajectory to reach the surface before subsequently travelling laterally along the surface for collection at the $n^{++}$ bias ring.

It is essential to highlight that both the time stamp, understood as the temporal delay for the signal to cross a predetermined signal threshold, and the overall collection time have to be intrinsically dependent upon the strength of the electric field perpendicular to the surface and the distance from the $n^{++}$ bias ring. Furthermore, charge carrier mobility emerges as a critical factor influencing the collection time, with its value dependent on the electric field strength, bulk resistivity, and the charge carrier type, whether electrons or holes^26^ depending on each given substrate material (here single crystal Si).

### Experimental methods

#### Transient current technique

The Transient Current Technique (TCT) has long been a foundational and versatile tool for the comprehensive investigation of semiconductor devices, a testament to its enduring utility in scientific research^27,28^. With TCT a pulsed laser system, with which one can tune the temporal and rate parameters, is used. This laser system serves as the primary excitation source, inducing the controlled generation of electron-hole (e-h) pairs within the semiconductor material, with a particular emphasis on silicon^29^.

**Fig. 3:**
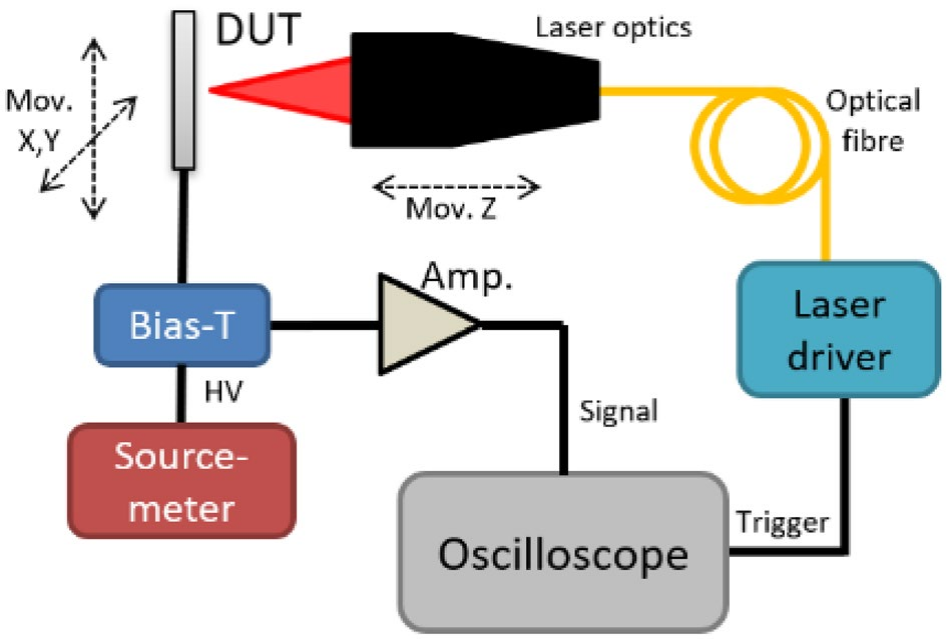
Schematic of the Transient Current Technique set-up^33^.

Figure 3, provided courtesy of Particulars^30^, provides a schematic representation of our experimental configuration. The detector under test (DUT) is illuminated with a blue laser, operating at a wavelength of 404 nm. Our laser system has the capability to achieve a beam diameter characterized by a full width at half maximum (FWHM) of less than 20 $\mu$m. Furthermore, the laser pulses themselves are distinguished by their temporal characteristics, spanning a duration ranging from 350 to 4000 ps, with pulse power levels similar with those generated by a few minimum ionizing particles (MIP). It should be noted that this wavelength corresponds to an absorption depth of approximately 120 nm within the silicon substrate, i.e all photons are absorbed close to the surface.

The output response of this controlled laser-induced excitation is inherently transient in nature. To effectively manage and disentangle this transient signal from the persistent direct current (DC) component, we employ a Bias-T configuration. Subsequently, the transient signal undergoes amplification, by a Cividec TCT Amplifier with a gain of 44.9 dB and a bandwidth of 2.9 kHz to 2 GHz^31^. The amplified transient signal is then acquired and read out with a DRS4 oscilloscope^32^. The signal is synchronised with the laser by connecting the output trigger of the laser diode to the oscilloscope.

A feature of our experimental configuration is its capacity to perform two-dimensional position scans, a capability facilitated by precision x-y movable stages. This functionality allows us to conduct in-depth investigations into the detector’s response as a function of laser beam position. Consequently, we gain valuable insights into the intricate spatial dependence characteristics inherent to the device under test.

The data acquisition and control of all of the constituent elements within our experimental setup are controlled by a LabVIEW program, provided by Particulars^30^.

### Responsitivity measurement setup

**Fig. 4:**
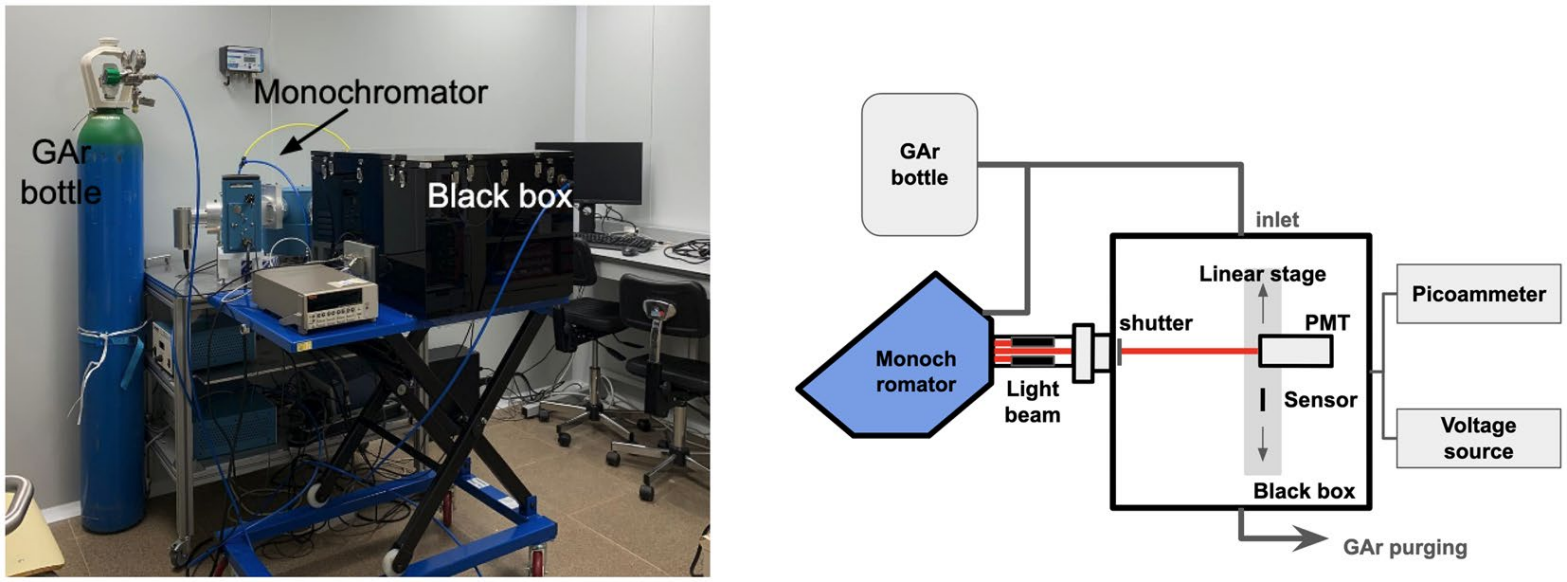
**Left:** View of the VUV setup at IFIC. **Right:** Diagram of the VUV measurement setup.

An experimental setup to characterise photosensors under controlled conditions of wavelength, atmosphere, and illumination was used which puts particular emphasis on the operation in the vacuum ultraviolet (VUV) range. Shown in Fig. 4, is permanently installed and available at the Instituto de Física Corpuscular (IFIC, Spain).

The experimental setup is built around a McPherson Model 234/302 0.2 m VUV monochromator, which provides a spectral resolution of 0.1 nm. The monochromator is coupled to a collimator and a custom black enclosure made of polymethyl methacrylate (approximately 60 cm $\times$ 50 cm $\times$ 50 cm), enabling measurements under light-tight conditions. The light source consists of two interchangeable lamps: a deuterium lamp covering the 100–300 nm range and a tungsten lamp for the 300–550 nm range. A mirror system allows rapid switching between the two sources. Both the monochromator and the enclosure are continuously purged with high-purity argon gas (99.999%, ALPHAGAZ 1) in order to ensure transmission in the vacuum ultraviolet (VUV) region, where air is otherwise opaque. All measurements are performed in a clean, dark laboratory environment.

The black enclosure is instrumented with temperature and humidity sensors and a motorised linear stage that enables precise positioning and alignment of the devices under test with respect to the incoming beam. A remotely operated shutter allows the beam to be blocked for the acquisition of dark current signals. The detectors are powered with a CAEN NDT1470 high-voltage supply, and the photocurrents are read out with a Keithley 8485 picoammeter. All elements of the setup are integrated into an automated control framework consisting of an Arduino board and custom Python scripts.

Before data taking, the system is purged with argon until a gas purity level is reached that guarantees VUV transmission. Within the enclosure, the linear stage alternates between a calibrated photomultiplier tube (Hamamatsu PMT R6836) and the sensor to be characterised. The measurement cycle begins with a wavelength scan using the reference PMT, followed by a scan with the device under test, and is completed with a second PMT scan. This redundancy accounts for possible variations in signal transmission due to changes in gas purity during the run. For each sensor and wavelength, the procedure involves first recording the dark current with the shutter closed, followed by the signal measurement with the shutter open, thereby correcting for temporal drifts in the dark current.

## Results

### Electrical characterisation

**Fig. 5:**
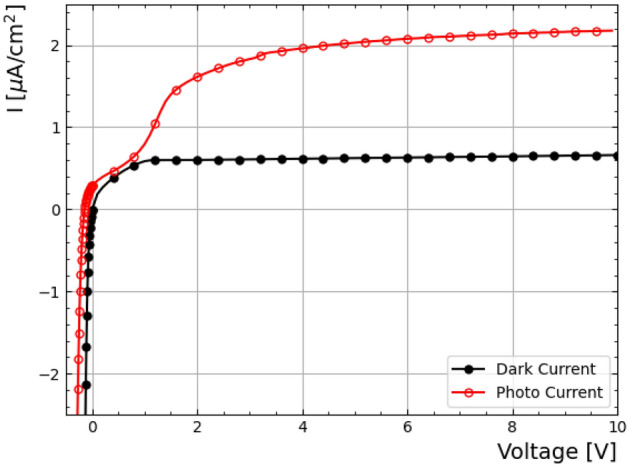
Current vs voltage (IV) characteristics measured using a Keithley 2410 source meter under dark (black) and illuminated (red) conditions. Positive voltage corresponds to reverse bias, while negative voltage corresponds to forward bias.

Current vs voltage (IV) measurements were performed using a Keithley 2410 source meter, providing voltage sweeps in both forward- and reverse-bias configurations, where positive voltage corresponds to reverse bias. The sweep was conducted in 0.1 V increments in the reverse bias regime (up to +10 V) and in 0.01 V increments in forward bias. Figure 5 shows the data and photocurrent response of the IV,. In dark conditions, the IV characteristics display the expected rectifying behaviour of a diode. Under illumination, the forward bias region shows the anticipated increase in current as well as an increase in the breakdown voltage, reflecting the influence of photogenerated carriers on the device’s conduction characteristics. In reverse bias, a rise in photocurrent is observed up to approximately +2 V, consistent with the charge vs voltage behaviour shown in Fig. 7 and indicative of the device reaching full depletion. The device, after full depletion, has a leakage current of 600 nA/$cm^2$. All measurements were performed at room temperature and the illumination provided by a white light source. As shown in Fig. 5, the measured dark current is relatively high, exceeding 500 nA/$cm^2$, compared with typical silicon PIN diodes, which usually exhibit dark currents below 10 nA/$cm^2$^34–36^. In the present device, the graphene layer electrically bridges the guard and bias rings, preventing a clear separation of bulk and surface currents. The total dark current in a semiconductor diode can be understood as the sum of a bulk generation–recombination current, arising from trap-assisted carrier generation within the depletion region, and a surface or interface current, associated with traps or defects at the semiconductor surfaces or interfaces^37,38^.

### Waveform analysis with TCT

To evaluate the device’s performance characteristics, we performed a 2D scan, over the entire area of the detector’s active region in steps of 35 $\mu$m. At each point probed we recorded the induced current signal, providing valuable insights into both the charge collection and the temporal attributes of the signal, including its rise time, decay time and peak value^28^.

**Fig. 6:**
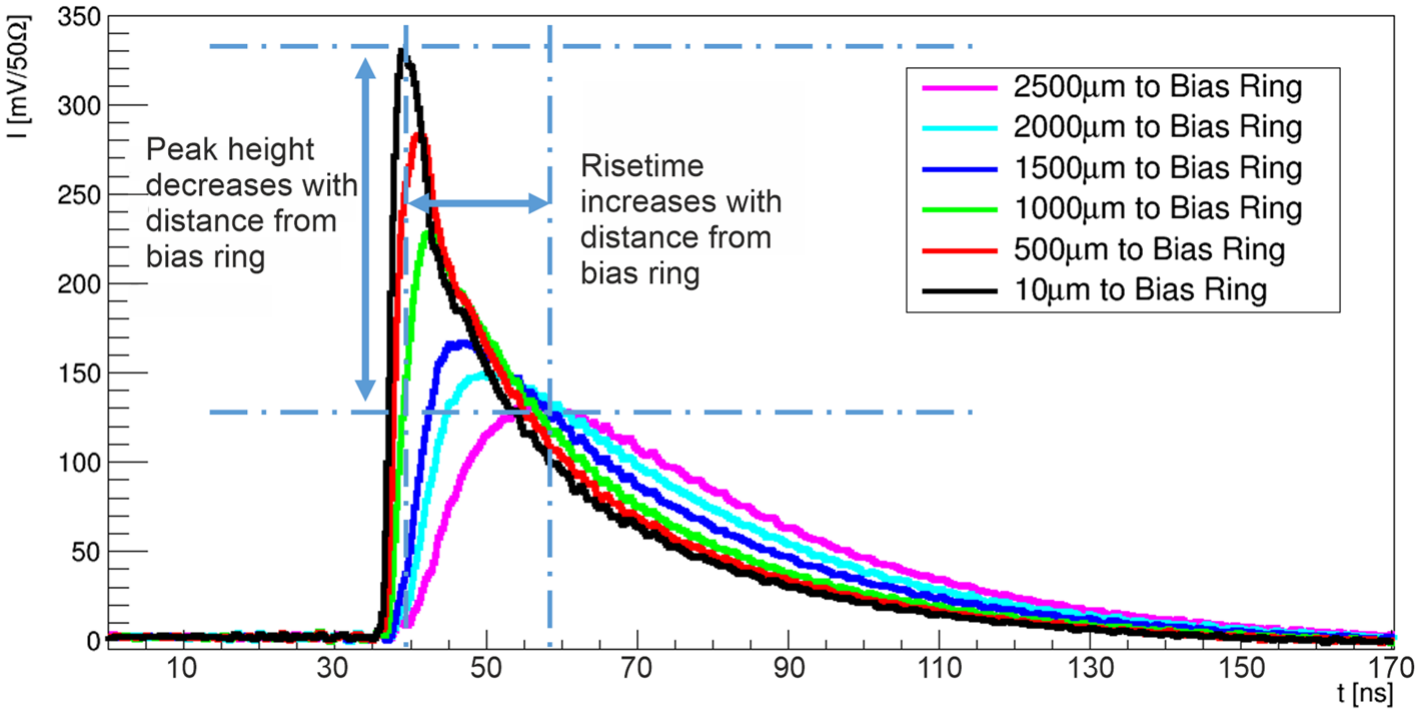
Experimental transient waveform response as a function of the position of the laser relative to the bias ring.

Understanding the total charge collected is of utmost importance for evaluating the charge collection efficiency, CCE, across the device. Figure 6 shows several plots of the induced transient waveforms concerning the laser’s incident position in relation to the bias ring. As expected, as the laser spot is moved further from the bias ring, the waveform changes. These changes result in a progressive reduction of the peak amplitude and a consequential increase in the rise time, ultimately translating to a delay of the overall response time. As the laser’s incident position moves from 10 $\mu$m to 1500 $\mu$m, quantitatively, the peak intensity diminishes by a factor approximating 50%, whereas the rise time increases fourfold. Moreover, the total collection time of the signal exhibits a corresponding increase across this spatial range.

This spatial dependence of the detector’s response unequivocally underscores the device’s operational principle, a phenomenon corroborated by the simulation and depicted in Fig. 2. In brief, the charges generated within the bulk of the semiconductor substrate must traverse the resistive layer, just below the surface of the device, before they can be collected within the bias ring. The duration of this charge collection process, as reflected in the rise time and total collection time, is intrinsically proportionate to the spatial separation and the electric field intensity prevalent within the detector^26,39^. It is important to note that the total charge collected is uniform across the device.

**Fig. 7:**
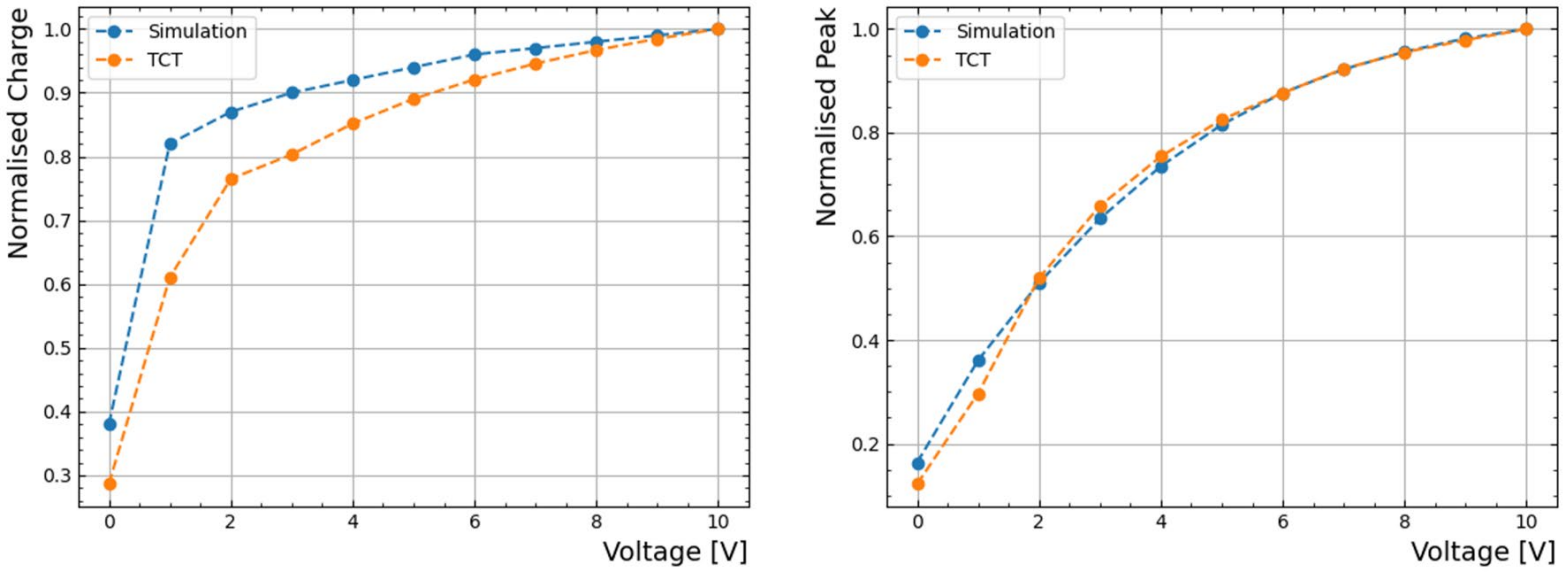
Normalised Photodetector response upon TCT testing. **Left**) Normalised charge vs voltage comparison of experimental and simulation results, **Right**) Normalised peak vs voltage comparison of experimental and simulation results.

Figure 7 shows the the normalized charge and peak response characteristics with respect to varying applied voltages. The charge and peak values are normalised to values obtained at 10 V. They show results obtained from both simulation and experimental data using the TCT, considering the laser focal point positioned at the device’s centre.

Figure 7 shows the simulation’s capacity to accurately predict voltage-dependent responses, in close agreement with the experimental TCT data. Evidently, the amplitude of the peak response increases with higher applied voltages, yielding a faster response. This trend, however, leads to saturation at approximately 10 V, which can be attributed to a limit in charge carrier velocity, a behaviour also reflected in the simulation. Additionally, the simulation effectively characterizes the lateral distribution of the electric field across the device’s surface.

However, the main result is related to the charge collection behaviour as a function of applied voltage, as illustrated in Fig. 7. It shows that, at lower voltages (< 1 V), the detector’s depletion remains incomplete, resulting in a comparatively modest charge collection, amounting to less than 60% of the total charge. In contrast, within the voltage range of 2-10V, charge collection demonstrates a nearly linear ascent, culminating at the maximum applied voltage of 10V. The reduction in CCE at lower voltages can be explained by the slow motion of charge carriers under weak electric fields, leading to a heightened probability of charge recombination within both the bulk and along the surface.

The waveform analysis highlights the importance of operating the detector at the highest possible (or high enough) bias voltage. Such an operational strategy ensures the collection of the maximum achievable charge, offering the added advantage of faster charge collection, elevated signal amplitudes, heightened signal-to-noise ratios, and reduced collection times.

### Extraction of the electric field profile - delayed peak method

If e-h pairs are generated far from the bias ring but close to the surface, as with a blue laser with a penetration of a few hundred nanometers, the electrons generated simply drift in line with the field parallel to the detector surface in the direction of the bias ring. The holes generated drift to the backside of the detector (35 $\mu$m), assuming the detector is fully depleted. As the lateral length of the detector is $\approx$ 2 orders of magnitude larger than the thickness, the rise time ($t_{r}$) dependence of the signal can be attributed to the drift of the electrons while the hole contribution can be neglected. This approximation holds for measurements made at distances greater than the thickness of the detector from the bias ring.

Consistently, a change in the laser position (probed point) results in a change of the rise time. The difference in rise time corresponds to the change in the drift length of the electrons. Therefore, the drift velocity ($v_e$) of the electrons can be calculated from the dependence of the rise time on the laser position. To calculate the electric field (E) one must take into account the change in the peak value, as shown in Fig. 6, the electron mobility ($\mu _{e}$) and E(x) can be calculated using^40^:

$$v_e(x) = \frac{\Delta x}{\Delta t_{r}} = \mu _{e}(E)E(x)$$

**Fig. 8:**
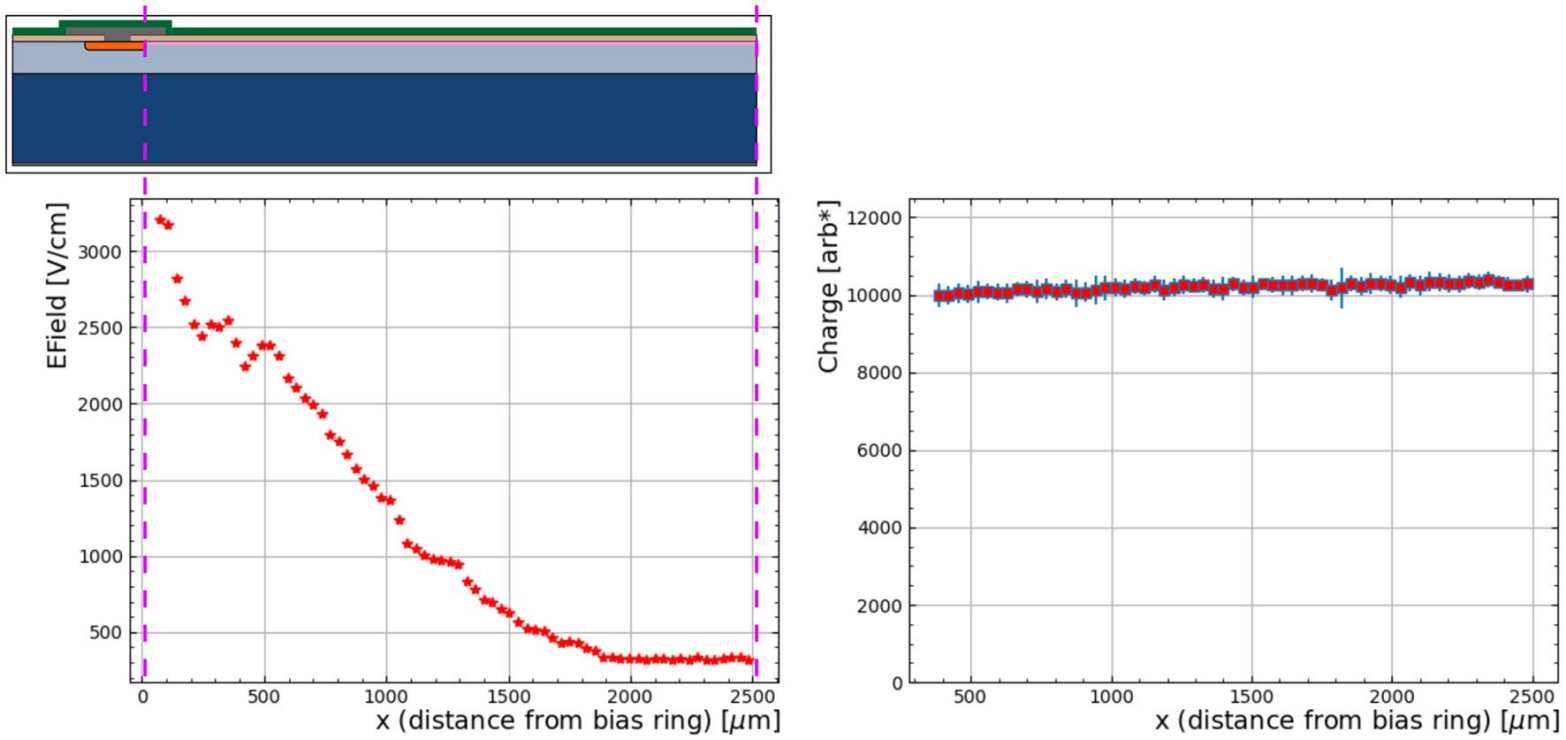
**Left**) Average electric field as a function of position from the bias ring at 30 V. **Right**) Charge collection as a function of distance from the bias ring.

Figure 8 shows the calculated lateral electric field as a function of the laser position at a reverse bias of 30V. As expected the electric field reduces as we move further from the bias ring. As the electric field reduces the drift velocity reduces and the probability of recombination increases and could be a reason for the drop in charge at low bias voltages as shown in Fig. 7.

At a bias voltage beyond that of the saturation shown in Fig. 7 the charge collected across the 5 mm-wide detector is significantly uniform as shown in Fig. 8. This is a key feature of this device. The plot shows the charge collected, at 30V, as a function of distance from the bias ring. Uniformity is one of the relevant characteristics featuring our GoS devices, as it is critical for imaging applications to have a uniform response independent of interaction position^41^.

### Responsivity

**Fig. 9:**
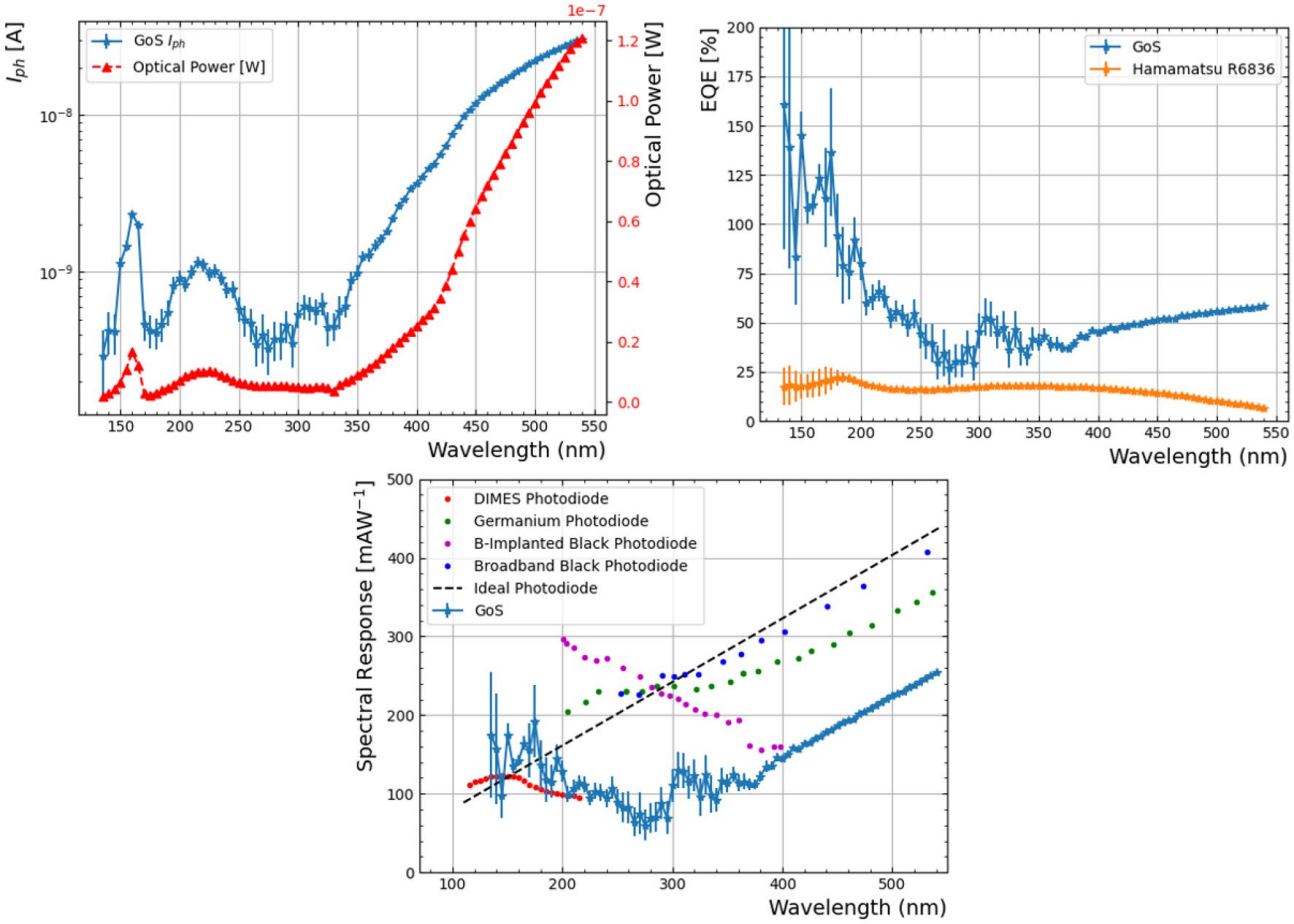
**Top Left**) Measured photocurrent as a function of the wavelength for the IMB-CNM GoS detector and calibrated incident optical power. **Top Right**) External quantum efficiency as a function of the wavelength for the IMB-CNM GoS detector and the reference PMT R6836 from Hamamatsu. **Bottom**) Spectral response as a function of the wavelength. This is a comparison of the spectral response for the GoS device alongside the state of the art devices. The data has been extracted using https://plotdigitizer.com/app from the references^11,12,43,44^ respectively.

To evaluate the sensing performance of the GoS device, the acquired data was analysed following a normalisation procedure based on the incident photon flux. For each wavelength, the net current measured by the sensor (after subtraction of the dark current) was divided by the incident light intensity, previously determined from the calibration curve of a reference PMT R6836 provided by Hamamatsu. Since the GoS device operates without internal gain, this approach directly yields its quantum efficiency. Each current value corresponds to the average of 50 acquisitions, with the associated uncertainty given by the standard deviation, which is a qualitative indicator of the electronic noise of the system. To determine the incident light, the mean of the two PMT measurements (before and after the sensor acquisition) was used, so that the error also accounts for possible fluctuations in light intensity during the run. The results are presented in Fig. 9(right), where the error bars are seen to clearly increase in the vacuum ultraviolet (VUV) range (< 200 nm). This larger uncertainty originates from fluctuations in the transmitted light, induced by variations in argon purity that affect transparency in this wavelength region.

The photocurrent, responsivity and external quantum efficiency (EQE) of the GoS device were measured across the wavelength range of 120–540 nm, as presented in Fig. 9 (top left, bottom and top right, respectively). These results are compared with state-of-the-art photodiodes previously discussed in the introduction. The comparison data were extracted from published plots using the online tool *PlotDigitizer* (https://plotdigitizer.com/app). The GoS responsivity was determined relative to a calibrated PMT R6836, as described in the Methods section. The measurement uncertainty is notably higher in the 120–350 nm range compared to longer wavelengths. This is primarily attributed to the reduced SNR at shorter wavelengths, where the incident light intensity is significantly lower. In the 200–540 nm region, the GoS device exhibits responsivity values of 100–200 $mA W^{-1}$, which are approximately 1.5 to 2 times lower than those reported for similar devices in the literature. Nevertheless, its qualitative performance remains close to that of an ideal photodiode. As a reference, in the 250–540 nm wavelength range, the boron-implanted black silicon photodiode^12^ demonstrates an almost ideal response, with responsivity values between 200 and 400 $mA W^{-1}$; however, its performance below 200 nm has not yet been reported. At shorter wavelengths (120–200 nm), only the DIMES photodiode has been previously reported, exhibiting excellent performance exceeding the ideal photodiode limit, with responsivity values above 100 $mA W^{-1}$ at 120 nm. In this range, the GoS device demonstrates a comparable, and in some instances slightly superior, response, though the associated measurement uncertainty remains substantial.

The relative EQE, calculated against the calibrated PMT R6836 reference (Hamamatsu), is shown on the top right side of Fig. 9. An ideal photodiode is expected to exhibit an EQE of 100%. The GoS device achieves EQE values close to or slightly above 100% in the 120–200 nm range, decreasing to approximately 50% across the 200–540 nm range. The EQE values with responses above 100% for high-energy photons, low wavelength, can be explained by secondary ionization^42^.

## Summary

A scalable semiconductor device featuring a minimal dead layer at the entrance window has been successfully designed and fabricated at the IMB-CNM. This achievement is attributed to the smart integration of graphene in combination with a dielectric, acting as a transparent field plate. The evaluation of this innovative device concept and performances was based on the (TCT) to investigate both local and global responses.

The outcomes of this study revealed a direct correlation between the collected charge and the applied bias voltage, particularly beyond the full depletion. It was observed that the loss of charge at low bias voltages was primarily due to recombination events occurring along the device’s surface. Yet, importantly the device exhibited a **uniform charge response** across the entire detector surface, although there was a noteworthy reduction in peak amplitudes and an increase in rise times as one moved further away from the bias ring.

Significantly, our device boasts the capability of achieving full depletion beneath the region covered by the graphene layer, setting it apart from previously discussed designs^13,19–21^. Consequently, this critical feature opens up opportunities for the segmentation of the detector from the backside, enabling the creation of an imaging sensor based on this our novel architecture.

Spectral response could be performed over the wavelength range of 120–540 nm. The results demonstrate that the device exhibits a response comparable to, or marginally superior to, previously reported devices in the VUV region. When compared with other induced-junction photodiodes, the device shows a slightly reduced response in the DUV range but an enhanced response at wavelengths below 200 nm. Increased measurement uncertainty was observed under low-light conditions, which can be attributed to a relatively high leakage current. This current is likely associated with surface charge accumulation, typically mitigated by isolating the surface through a guard ring connection. In the present design, however, the graphene layer electrically bridges the guard ring and the bias ring, thereby preventing the separation of bulk and surface currents. As a design technology fault, this issue can be addressed by simply adjusting detector architecture in future generations of the GoS device. In future versions of the detector, we will perform a more systematic assessment of the robustness of the graphene/high-k stack. This will include cumulative VUV dose studies and pre-/post-exposure comparisons of EQE and IV characteristics on new samples to evaluate potential drift or degradation. Additionally, we plan to investigate the device response under low-energy X-ray illumination, as this represents a promising avenue for extending the detector’s applicability.

Building upon our extensive expertise in fabricating fine-pitch pixelated detectors and combining it with this technology, we are well positioned to propose and develop an imaging detector featuring an entrance window with minimal dead zones. Such a device holds immense promise not only for applications in the deep ultraviolet (DUV) range but also for “soft” X-ray imaging at synchrotron light sources, particularly within the “water-window” energy range (approximately 500 eV)^45^.

## Data Availability

The data that support the findings of this study is available from the corresponding author on reasonable request.
